# A Novel Animal Model of Partial Optic Nerve Transection Established Using an Optic Nerve Quantitative Amputator

**DOI:** 10.1371/journal.pone.0044360

**Published:** 2012-09-04

**Authors:** Xu Wang, Ying Li, Yan He, Hong-Sheng Liang, En-Zhong Liu

**Affiliations:** 1 Department of Neurosurgery, First Affiliated Hospital, Harbin Medical University, Harbin, China; 2 Department of Neurosurgery, NO.211 Hospital of PLA, Harbin, China; 3 Department of Nutrition and Food Hygiene, Public Health College, Harbin Medical University, Harbin, China; 4 Department of Pathology, Basic Medical College, Harbin Medical University, Harbin, China; Pomona College, United States of America

## Abstract

**Background:**

Research into retinal ganglion cell (RGC) degeneration and neuroprotection after optic nerve injury has received considerable attention and the establishment of simple and effective animal models is of critical importance for future progress.

**Methodology/Principal Findings:**

In the present study, the optic nerves of Wistar rats were semi-transected selectively with a novel optic nerve quantitative amputator. The variation in RGC density was observed with retro-labeled fluorogold at different time points after nerve injury. The densities of surviving RGCs in the experimental eyes at different time points were 1113.69±188.83 RGC/mm^2^ (the survival rate was 63.81% compared with the contralateral eye of the same animal) 1 week post surgery; 748.22±134.75 /mm^2^ (46.16% survival rate) 2 weeks post surgery; 505.03±118.67 /mm^2^ (30.52% survival rate) 4 weeks post surgery; 436.86±76.36 /mm^2^ (24.01% survival rate) 8 weeks post surgery; and 378.20±66.74 /mm^2^ (20.30% survival rate) 12 weeks post surgery. Simultaneously, we also measured the axonal distribution of optic nerve fibers; the latency and amplitude of pattern visual evoke potentials (P-VEP); and the variation in pupil diameter response to pupillary light reflex. All of these observations and profiles were consistent with post injury variation characteristics of the optic nerve. These results indicate that we effectively simulated the pathological process of primary and secondary injury after optic nerve injury.

**Conclusions/Significance:**

The present quantitative transection optic nerve injury model has increased reproducibility, effectiveness and uniformity. This model is an ideal animal model to provide a foundation for researching new treatments for nerve repair after optic nerve and/or central nerve injury.

## Introduction

In mammals, the optic nerve, which consists of retinal ganglion cell (RGC) axons, is part of the central nervous system (CNS) [1]. RGCs are the output neurons relaying visual signals from the eye, extending along axons via the optic nerve to the brain [2], [3]. Traumatic optic neuropathies often lead to retrograde degeneration and apoptosis of RGCs. This can lead to visual impairment [4] and neurological dysfunction [5], [6]. Establishing an effective animal model of optic nerve injury is important to better understand the mechanisms behind RGC degeneration and neuroprotection, and further explore new drug targets and treatment options, such as cell transplantation, for optic nerve functional recovery and regeneration [7], [8].

Currently, there are two major classifications of optic nerve injury models, incomplete and complete. Incomplete models include crush [9]–[11], stretch [12], [13] and cold injury models [14]. Incomplete models are difficult to ensure uniformity in the degree of optic nerve injury and quantify accurately [15]. They are also not suitable for topical drug administration during treatment. Furthermore, the mechanisms of injury are complicated. The primary injury causes damage to the optic nerve and vascular system, meanwhile, the secondary ischemic injury occurred due to optic nerve swelling in the canalis opticus. Additionally, it is difficult to conduct systematic research due to the use of complex instruments, long-term surgical procedures, difficulty in operation and extensive interference factors. The complete injury model involves a complete transection of the optic nerve [16], [17]. This model is easy to establish, reproducible and directly demonstrates the degree of optic nerve injury. Moreover, the complete injury model assures consistency in the degree of injury between different nerve axons and between different animals. The features of the complete injury model ensure fewer experimental errors. Furthermore, it is a sufficient model for examining nerve degeneration and apoptosis after injury. Additionally, the optic nerve transection model is suitable for evaluating treatment effects of topical drug administration, bridge grafting and neural transplantation. Therefore, the optic nerve axon transection model plays an important role in investigating the mechanisms behind degeneration and regeneration after CNS injury [18] and is a popular model for examining potential neuroprotective strategies *in vivo*. However, after complete transection the optic nerve is separated into two retracting nerve stumps that are unable to maintain the original integrity and anatomical position, thereby restricting neuroprotection and recovery *in situ* [3].

**Figure 1:**
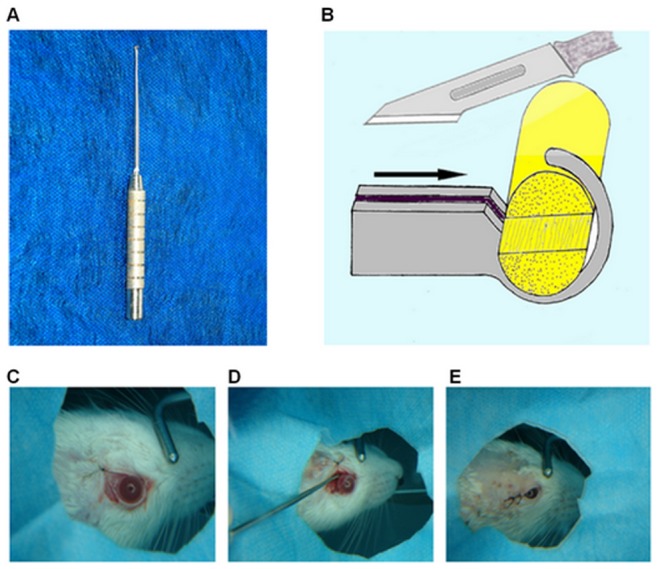
Optic nerve quantitative amputator working principle and application in rats. (A) The main parts of the amputator contains: frontal harness hole, cutting groove, connecting rod, and handle. (B) Illustration of the amputator working principle. Black arrow indicates the cutting direction. (C) Operational procedure of optic nerve semi-transection in rats: cutting apart the outer canthus, suspending and fixing the palpebra superior; (D) Exploring the optic nerve, and transecting the superior portion of optic nerve using the amputator; and (E) Suturing the conjunctiva and skin, finally, antibiotic ointment applied to the wound.

In the present study, we created a novel instrument for establishing a partial optic nerve transection model, which we called the Optic Nerve Quantitative Amputator (Figure 1). Using this specially-designed instrument, the optic nerve can be transected quantitatively and reliably with good reproducibility. As there is good conservation between the rat and human genomes, and good anatomical similarity of the optic nerve, rats are widely used as a simple animal model for researching human optic disease and regeneration [19], [20]. This partial optic nerve injury model in rats is needed for investigating the differential mechanisms of primary and secondary neuronal degeneration [21], [22]. Moreover, it could establish a foundation for examining future neuroprotective strategies and help provide new medical interventions for promoting nerve regeneration following optic nerve and CNS injuries.

## Results

### RGC Degeneration after Rat Optic Nerve Semi-transection

**Figure 2:**
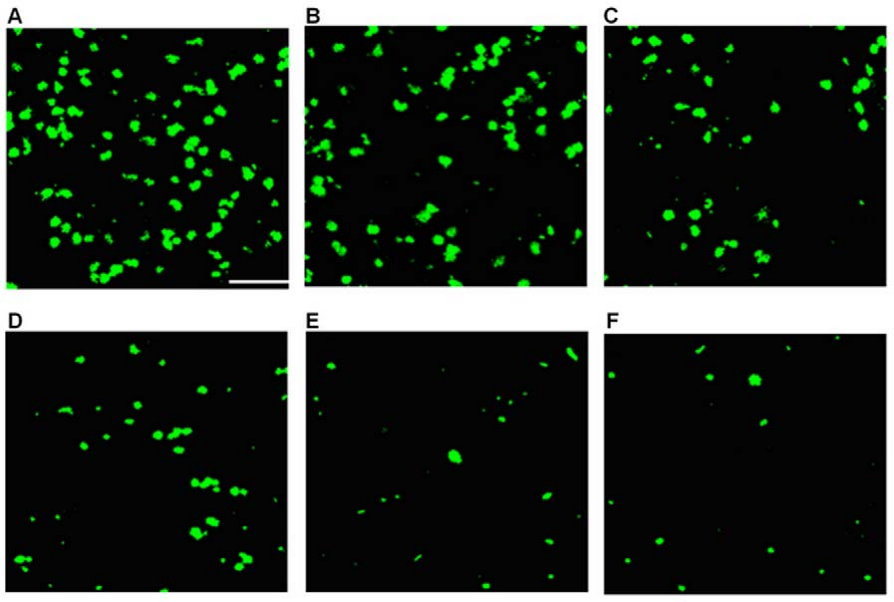
Representative photographs of flat-mounted retinas. (A) FG-labeled RGCs in corresponding regions of control retina; (B-F) Show that the FG-labeled RGCs in the optic nerve semi-transected model at different time-points: (B) 1 week, (C) 2 weeks, (D) 4 weeks, (E) 8 weeks, (F) 12 weeks. As shown above, the number of RGCs reduced over time after the semi-transection. Scale bar, 50 µm (White Line).

**Figure 3:**
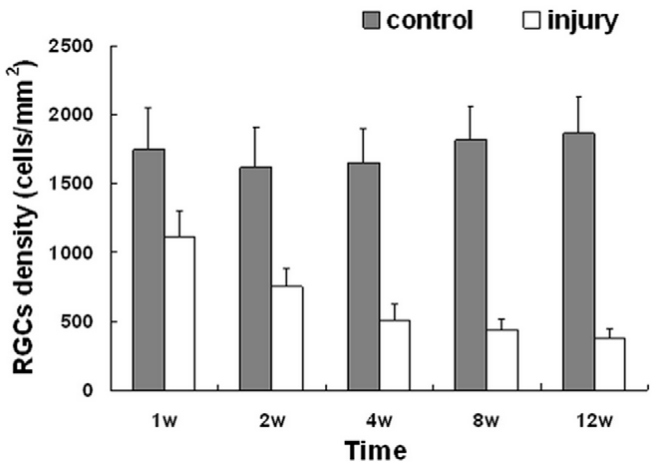
The density of RGC FG-labeled in the retinal between the control and experimental eyes. Data indicate as Mean ± SD (n = 6 for each group). There was no significant difference between different time points among the controls (P>0.05). The RGC density of experimental eyes were significantly less than the control eyes in the same rats at different time points (1 week, p<0.001; 2 weeks, p<0.001; 4 weeks, p<0.001; 8 weeks, p<0.001; 12 weeks, p<0.001). The RGC density decreased gradually over time after injury. Error bars indicate SD.

The results showed that the mean density of RGCs in the control eyes was 1741.23±104.33 cells/mm^2^ for all the control groups (n = 30). In the experimental eyes group, RGC survival decreased with time after injury (n = 6 for each group). The densities of remaining FG retro-labeled RGCs after optic nerve semi-transection were 1113.69±188.83 cells/mm^2^ (63.81% survival rate at week 1), 748.22±134.75 cells/mm^2^ (46.16% survival rate at week 2), 505.03±118.67 cells/mm^2^ (30.52% survival rate at week 4), 436.86±76.36 cells/mm^2^ (24.01% survival rate at week 8) and 378.20±66.74 cells/mm^2^ (20.30% survival rate at week 12). The mean rate of RGC apoptosis is highest in week 1, with 627.54 cells/mm^2^ per week undergoing apoptosis, following which it slowly decreased as follows: between weeks 1 and 2, the rate was 365.47 cells/mm^2^ per week; from weeks 2 to 4, the rate was 121.60 cells/mm^2^ per week; from weeks 4 to 8, the rate was 17.04 cells/mm^2^ per week; and from weeks 8 to 12, the rate was 14.66 cells/mm^2^ per week (Figure 2,3).

### Comparison of Optic Nerve Distribution after Optic Nerve Injury

**Figure 4:**
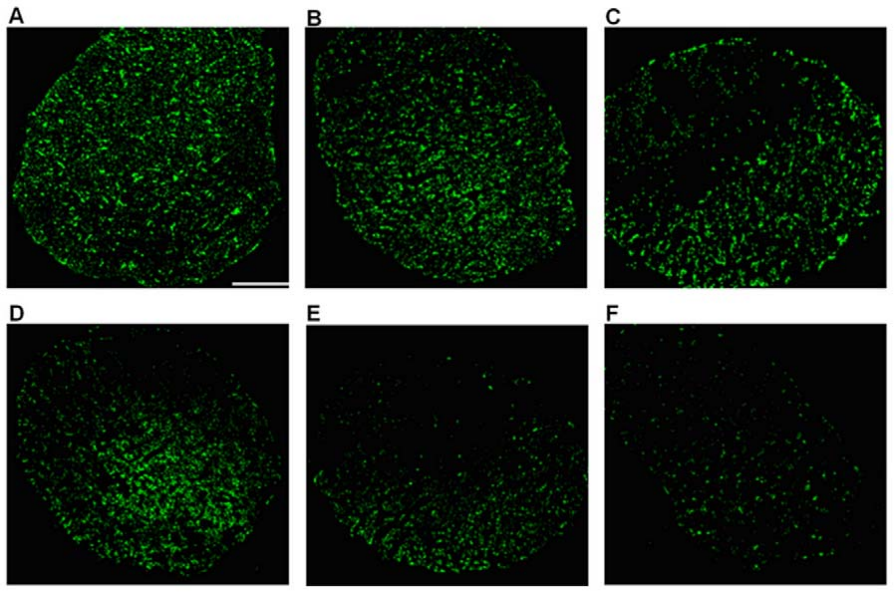
Representative photographs of optic nerves with FG-labeled optic nerve fibers. (A) The fluorescence of the optic nerve fibers was evenly distributed and abundant in the controls. (B–F) The number of optic nerve fibers labeled at the superior semi-transected section decreased over time after injury. (C–F) 2 weeks post injury, there was a decrease in the number of optic nerve fibers at inferior intact section of the optic nerve, in which the number of the labeled nerve fibers decreased gradually over time. (B = 1 week, C = 2 weeks, D = 4 weeks, E = 8 weeks and F = 12 weeks). Scale bar, 100 µm (White Line).

The FG-labeled specimen of the optic nerve fibers showed that the nerve fibers were evenly distributed and abundant in the control eye group. However, after optic nerve semi-transection, the nerve fibers were unevenly distributed and the number of fibers at the transected optic nerve site decreased gradually over time. Two weeks post injury, there was a decrease in the number of optic nerve fibers at the intact optic nerve section (Figure 4).

**Figure 5:**
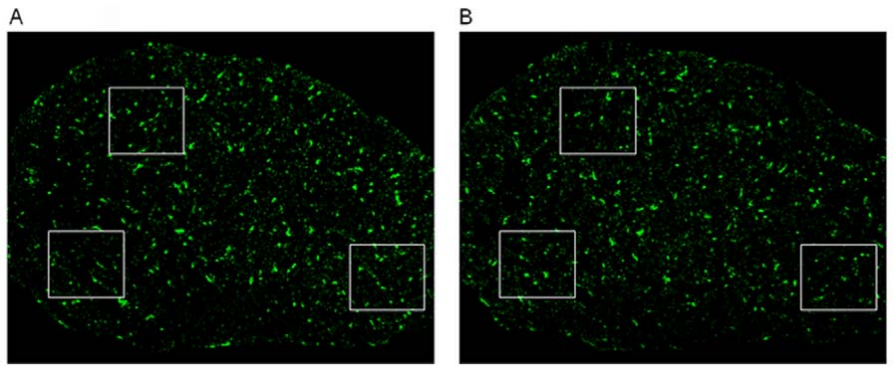
Observations of two consecutive slices from the same nerve in the control eye. (A) Slice near the eye. (B) Slice away from the eye next to (A). The distribution and fluorescence quantity were different between the two consecutive slices as indicated by White Square. Scale bar, 100 µm (White Line).

Meanwhile, observations of two consecutive slices from the same nerve 1.0 mm behind the eyeball in the control eye group were compared (Figure 5). The distribution and the number of FG-labeled optic nerve fibers were slightly different between two consecutive slices. The total amount of FG-labeled fibers was less than the total amount of FG-labeled RGCs in the retina.

### Comparisons of P-VEP after Optic Nerve Injury

**Figure 6:**
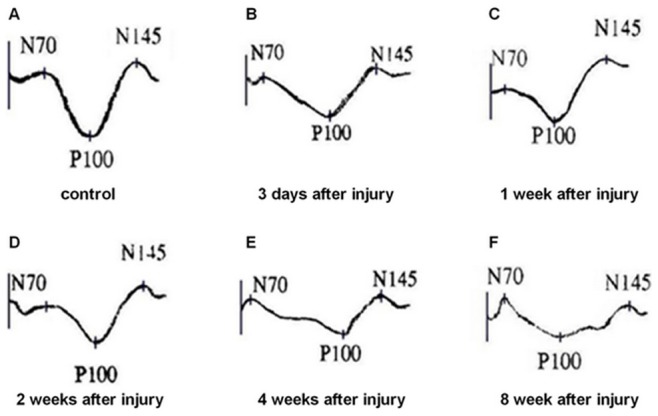
The N_70_-P_100_-N_145_ wave of P-VEP. (A) The latency of the N_70_-P_100_-N_145_ wave was short and the electric potential amplitude was steep in the control eyes. (B–F) The N_70_-P_100_-N_145_ wave became delayed and decreased after semi-transection of the optic nerve.

Results show that each peak of P-VEP could be distinguished clearly in the control eyes of rats and the N_70_-P_100_-N_145_ waveform latency was short and the electric potential amplitude was steep. The N_70_-P_100_-N_145_ waveform gradually returned to the baseline, the latency was obviously prolonged and the amplitude was significantly reduced after semi-transection of the optic nerve (Figure 6).

### P_100_ Wave Latency

**Figure 7:**
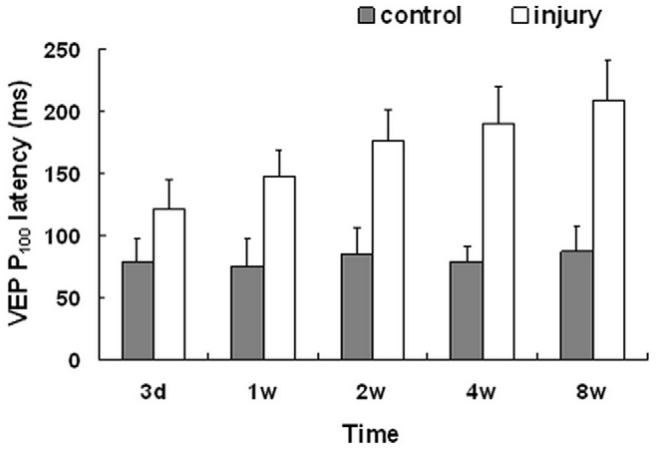
P_100_ wave latency of P-VEP. There was no difference in P_100_ wave latency at different time points among the control eyes (P>0.05, n = 6/group). Compared with the control, the latency of the P_100_ wave was significantly lengthened in the experimental eye groups at the same time point after optic nerve semi-transection (P_3d_<0.01, P_1w_<0.001, P_2w_<0.001, P_4w_<0.001, P_8w<_0.001, n = 6/group); and the latency of the P_100_ wave lengthened gradually as time prolonged. Error bars indicate SD.

There was no difference in P_100_ wave latency among the V3D to V8W control eye groups (P>0.05, n = 6/group). Compared with the control, the main P_100_ wave latency was significantly lengthened in the experimental eye groups at the same time point among the V3D - V8W (P<0.01, n = 6/group), and the main P_100_ wave latency lengthened gradually over time (Figure 7). The increasing ratio of P_100_ wave latency became greater with time extension after optic nerve injury, with an obvious increase from day 3 to week 4, which increased from 54.27% on day 3 to 95.99% at week 1. After week 1, the ratio increased from 108.13% at week 2 to 139.98% at week 4, during which the latency increased relatively quick. The increasing trend of latency maintained smooth from weeks 4 to 8 and the increasing ratio reached a peak of 140.26% at week 8.

### N_70_-P_100_ and P_100_-N_145_ Amplitudes

**Figure 8:**
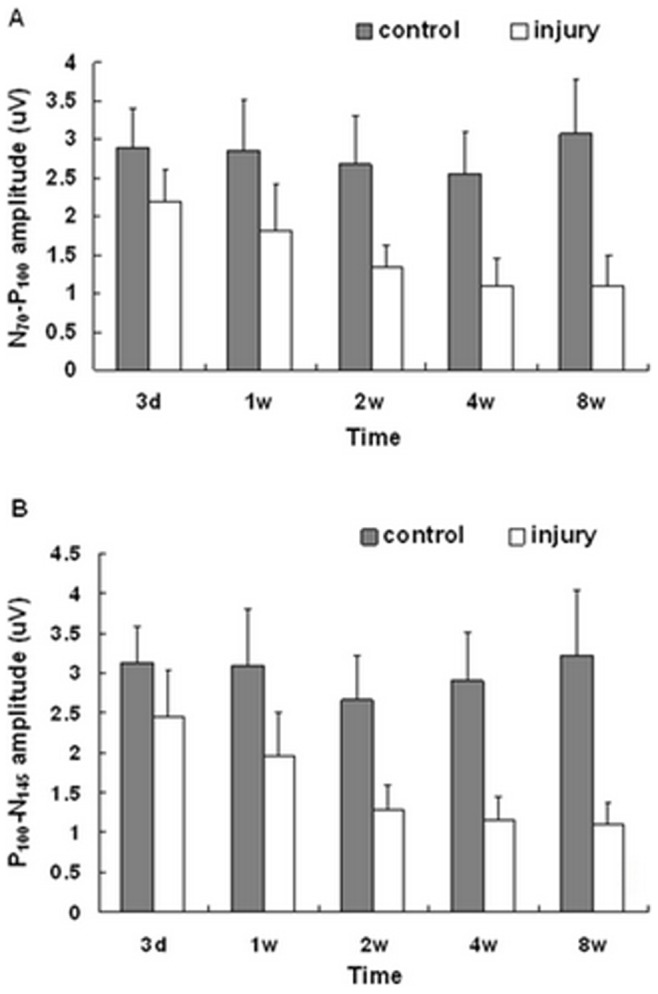
N_70_–P_100_ and P_100_-N_145_ amplitudes of P-VEP. (A) The N_70_–P_100_ amplitudes significantly decreased after optic nerve semi-transection compared with the control eyes at the same time point (P_3d_<0.05, P_1w_<0.01, P_2w_<0.001, P_4w_<0.001, P_8w_<0.001, n = 6/group). (B) The P_100_-N_145_ amplitudes significantly decreased after the optic nerve injury compared with the control groups at the same time point (P_3d_<0.05, P_1w_<0.01, P_2w_<0.001, P_4w_<0.001, P_8w_<0.001, n = 6/group), the amplitudes tendency of both (A) and (B) decreased gradually as time prolonged in the experimental groups individually. Both (A) and (B) amplitudes have no significant difference at different time points among the control groups. (P>0.05, n = 6/group). Error bars indicate SD.

There was no difference in N_70_-P_100_ and P_100_-N_145_ amplitudes of P-VEP at different time points among the control eye groups (N_70_-P_100_, P>0.05, P_100_-N_145_, P>0.05, n = 6/group). Compared with the control, the N_70_-P_100_ and P_100_-N_145_ amplitudes of the experimental eye groups were significantly decreased at the same time point (*P*<0.05, n = 6/group) (Figure 8).

### Pupil Diameter Response to Pupillary Light Reflex

**Figure 9:**
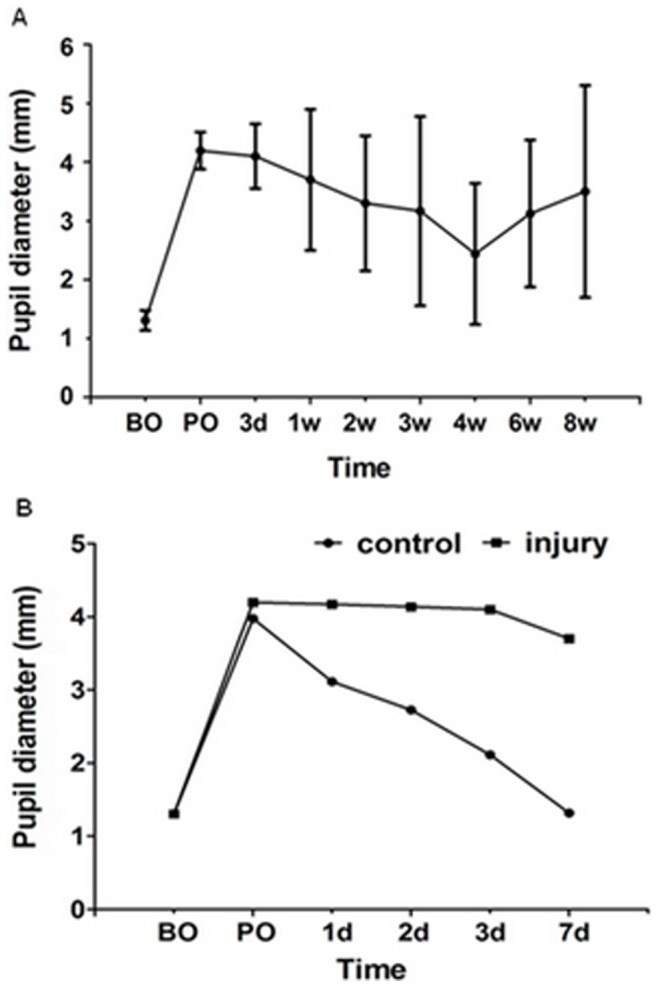
Pupil diameter response to pupillary light reflex. (A) The pupil diameter began to dilate significantly and immediately after optic nerve semi-transection compare with the control (P<0.001, n = 12), followed by gradual reduction until the week 4, corediastasis reappeared from the week 4 to week 8. Error bars indicate SD. (B) Corediastasis of both eyes were observed after injection of FG into the bilateral superior colliculi and lateral geniculate body. The pupil diameter of the control eyes recovered to normal size within 1 week, but the experimental eye did not recover. (BO is before operation; PO is post operation).

The pupil diameter in both eyes before semi-transection was 1.31±0.17 mm (n = 24) in the V8W and F12W groups. Corediastasis occurred after semi-transection of the optic nerve, with the pupil diameter immediately dilating to 4.20±0.31 mm and maintaining this size for the first three days after surgery. The pupil diameter was 4.10±0.55 mm on day 3, with a gradual reduction until week 4 (3.70±1.20 mm at week 1, 3.30±1.15 mm at week 2, 3.17±1.61 mm at week 3 and 2.44±1.20 mm at week 4). The pupil diameter dilated again from weeks 4 to 8 (3.13±1.25 mm at week 6 and 3.50±1.80 mm at week 8). Pupil diameter significantly dilated at different time points after injury compared with pre-surgery values (*P*<0.001, n = 12) (Figure 9A). In addition, corediastasis of both eyes was observed in the F1W group after injection of FG into the bilateral superior colliculi and lateral geniculate body. The pupil diameter of the control eye recovered to normal within 1 week, but the experimental eye did not recover (Figure 9B).

### The Correlation Coefficient of Parameters

**Figure 10:**
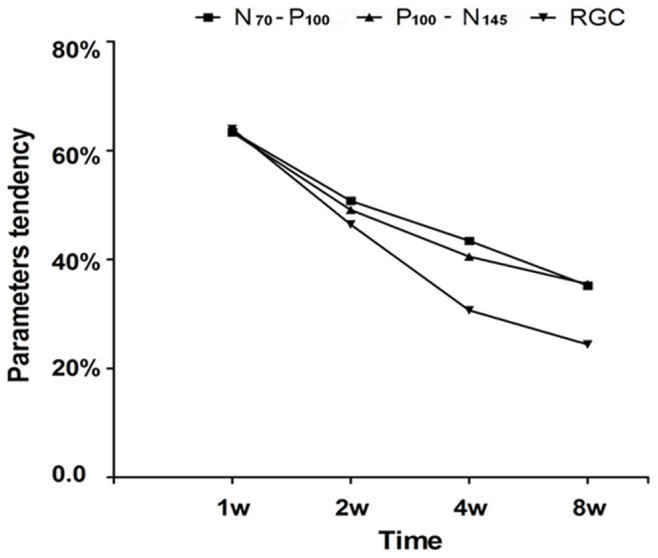
Correlation coefficient of parameters. The variation tendency of the RGC survival rate comparing with decreasing ratio of N_70_-P_100_ and P_100_-N_145_ amplitudes in the experimental eye groups at the same time point, the curve lines indicated that have significant similar tendency. These parameters correlate with each other.

The variation tendency of the RGC survival rate in the experimental eyes at week 1, 2, 4 and 8 groups were observed and compared with the variation tendency of the increasing ratio of P_100_ latency and decreasing ratio of N_70_-P_100_ and P_100_-N_145_ amplitudes at the same time point. We found that all four parameters correlate with each other. The RGC survival rate and the increasing ratio of P_100_ latency have a strong negative linear correlation (r = −0.9695); the RGC survival rate and the decreasing ratio of N_70_-P_100_ amplitude have a strong positive linear correlation(r = 0.9942); and the RGC survival rate and the decreasing ratio of P_100_-N_145_ amplitude have a strong positive linear correlation (r = 0.9917) (Figure 10).

## Discussion

### Operational Procedure

The main characteristics of the present operational procedure include: 1) A lateral canthotomy was adopted to decrease side effects, hemorrhage and mortality. This was chosen for three reasons: first, a 5.0 mm skin incision is minimally invasive; second, the optic nerve is revealed and explored through the corridor between the superior rectus and lateral rectus, without cutting off the superior rectus; third, it avoids an open excision of the orbital wall bone and damage of its periost, which can decrease the side effects of surgery, reduce subsequent infection, inflammation, hemorrhage and swelling of local soft tissue, which may compress the optic nerve. Furthermore, the operational procedures of canthotomy are simple, time-saving and without the requirement for special surgical instruments. 2) By suspending the palpebra frontalis outward and upward, the surgical visual field is revealed sufficiently. 3) The incision in the optic nerve meninges was only 2.0 mm, which maintained the integrity of the meninges of the optic nerve and preserved the internal environment to prevent neuroma formation and allow local injection of therapeutic substances into the meningeal tube at the injury site [15], [23]. It is beneficial to the oriented growth and regeneration of optic nerve axons; and also to the elimination of possible adverse reactions from substance diffusion to the back of the eye.

### Optic Nerve Quantitative Amputator

The animal model of semi-transection of the optic nerve was established using a self-designed optic nerve quantitative amputator, which has not been reported to date. The main characteristics of the optic nerve quantitative amputator are as follows: 1) It can transect the superior optic nerve quantitatively, ensuring uniformity of injury between different samples. Previously, partial transection models could only be established roughly using simple operating instruments, which was difficult to transect the same portion of the optic nerve equally and to quantify the degree of injury between different animals [21]. 2) The harness hole of the quantitative amputator can fix the optic nerve in a stable position during the operation. This helps prevent accidental damage to retinal central vessels or other tissues due to nerve movement during optic nerve transection, which also decreases experimental error and adverse reactions. Furthermore, other advantages of the harness hole include: prevention of stretch injury when transecting the optic nerve; ensuring the vertical amputation of the superior part of the optic nerve and preventing deflexion from influencing the experiment; decreasing systematic error; and improving accuracy. 3) It is convenient for operating in deep and narrow spaces. There are more advantages when using the quantitative amputator in combination with lateral canthotomy, as it decreases the surgical incision and the extent of dissecting intraorbital soft tissues. It is also suitable for performing semi-transection through the incision where the optic nerve meninges are cut open. 4) The optic nerve quantitative amputator uses an operating knife to transect the nerve to avoid crush and contusion injuries caused by scissors.

In the present study, the semi-transected scale of the optic nerve quantitative amputator was applied in an animal model, which transected the superior half of the optic nerve. The depth of the cutting groove of the amputator could be adjusted to 1/3, 2/3 or 1/4 depth in diameter to cut the optic nerves at depths of 1/3, 2/3 or 1/4 diameter. In conclusion, the application of the amputator, which makes quantitative and uniform incisions the nerve, may serve as an effective instrument for establishing animal models for the research of optic nerve injury.

### Characteristics of the Optic Nerve Semi-transection Injury

The method applied in the present animal model is transecting the superior half of the optic nerve. This method retains the inferior portion of the optic nerve, which prevents separation after transecting the optic nerve completely, decreases nerve instability and provides an anatomical base for the connection *in situ* for axonal regeneration after injury [3]. Therefore, this model is very suitable for evaluating the effectiveness of focal substance administration, nerve scaffold bridging and cell transplantation. In addition, the semi-transection model is beneficial to the comparison of morphological changes and distribution of nerve fibers in injured and intact areas. Thus, it is an effective method to study the differential mechanisms of primary and secondary degeneration of RGCs [21], [22]. Furthermore, this method can eliminate systemic effects caused by retinal vessel injury, thereby reducing interferences to experimental results.

In this study, the self-control method is applied by observing RGC density, optic nerve fiber distribution, pupillary reflex modification and recording the visual electrophysiology of the P-VEP latency and amplitude on the control and injured eyes in the same rat. RGC density of the optic nerve semi-transection injury was much lower than that of the intact optic nerve at different time points. The survival rate of RGCs reduced gradually after semi-transection as animal survival time was prolonged, which was consistent with the research results of Berkelaar *et al*. [16]. The reduction in RGC density is used for monitoring the degree of injury and RGC apoptosis after optic nerve damage [24], [25].

The P-VEP test results revealed that after optic nerve semi-transection, the latency of the P_100_ wave prolonged gradually over time and the amplitude of the N_70_-P_100_ and P_100_-N_145_ also reduced gradually over time. As previously investigated, the latency reflects the function of nerve conduction and the amplitude reflects the receptive function of the macula lutea. Both of these parameters objectively reflect the degree of optic nerve injury [26]. After analyzing the correlation coefficient of parameters, a strong correlation was observed. The change in RGC density correlates with the variation in P-VEP latency and amplitude after semi-transection (Figure 10), meaning that the novel semi-transection animal model is stable and reproducible.

The modification of pupillary reflex was a physical sign of optic nerve injury [27], [28]. After optic nerve semi-transection, corediastasis immediately appeared and the pupil shrank gradually until week 4. However, corediastasis appeared again after week 4, which was probably due to the deterioration of the optic nerve by secondary injury or may be a result of apoptosis. As a result, the progression of the injury was not identical as the continually progressive deterioration in RGC density and P-VEP latency and amplitude. Corediastasis of both eyes was observed in all rats in the F1W group after retrograde labeling of FG and the pupil size of the control eyes returned to normal within 1 week, but the experimental eye never recovered (Figure 9B). This physical phenomenon demonstrates that the provisional injury occurred at the bilateral superior colliculi and lateral geniculate body when the FG was being injected and this injury should recover after several days. All of the results indicate that the optic nerve semi-transection model was consistent with the clinical variation characteristics of the optic nerve and/or retina after injury [1], [14], [28], [29].

According to previous reports, RGC death rate is approximately 50–70% at 1 week after optic nerve transection and increases to 85–95% at 2 weeks [15], [16], [23], [25], [30], [31]. The data of the present study showed that the decrease in RGC density was 36.19% at 1 week after semi-transection of the optic nerve, RGC death was similar to previous reports, indicating that apoptosis at week 1 was mainly due to the primary injury. This model transects only the superior portion of the optic nerve, leaving the inferior portion unaffected. This leads to a degeneration of 50% RGCs, unless secondary degeneration occurs. Interestingly, the results of the present study show a decrease in RGC density by 53.84% at 2 weeks post injury. The slices of optic nerve distribution at 2 weeks after semi-transection show that the number of optic nerve fibers decreased obviously at the transected superior portion and the reduction had also began in the intact inferior portion (Figure 4C–F). These data not only indicate that the rapid rate of RGC apoptosis that had occurred by week 2 was mainly due to the primary injury at the transected superior portion, but also demonstrates that the secondary injury began to occur at the intact inferior portion. And, the results of the decreasing density of RGC from week 4, the secondary injury may persist until at least week 12. Simultaneously, by comparing the distribution of FG-labeled optic nerve fibers, the fluorescence quantification in the intact inferior portion also showed a stronger decreasing tendency after week 2 as survival period prolonged (Figure 4C–F). As most RGCs were lost by 2 weeks after optic nerve transection [16], [17], [30], [31], secondary injury could be considered according to the rate of RGC apoptosis per week and the variation in cell density, reflecting further RGC apoptosis at the intact inferior portion 2 weeks after semi-transection. The secondary degeneration might be due to a self-propagating process that dead neurons spread damage to neighboring cells, which were not originally injured, leading to a more extensive damage than the primary injury or it might be mediated by the release of toxins from the degenerating fibers [32]–[35]. The partial transection model gives a better understanding of secondary degeneration as the location of the primary injury is clearly known and the secondary degeneration could appear in a different area from the primary injury. Therefore, this novel optic nerve semi-transection animal model can effectively simulate the pathological process of primary and secondary injury [15].

The results of the present study showed that the total fluorescence of normal optic nerve fibers retro-labeled with FG was less than the number of RGCs in the retina of adult Wistar rats reported by Levkovitch-Verbin, Danias, and Sievers [36]–[38]. Comparing the two consecutive slices of the same optic nerve of the control eye, it was found that the total fluorescence emission and local distribution were different (Figure 5). Therefore, based on the results of our research, it is recommended that the total RGCs should be found by counting FG-labeled RGCs in the retina rather than counting FG-labeled optic nerve fibers in the optic nerve slice.

An animal model of optic nerve injury was successfully established using a self-designed optic nerve quantitative amputator. The advantages of this model include: simple injury instruments and surgical procedure; minimal trauma to the experimental animals; single injury factor; and the uniformity of injury degree, nature and location. This quantitative transection optic nerve injury model has better reproducibility, effectiveness and uniformity.

In conclusion, the optic nerve semi-transection model reflects obvious clinical features of optic nerve injury by the observed indicators [1], [39]–[41], and it can effectively simulate the pathological process of primary and secondary injury after optic nerve injury. This is an ideal animal model to use as a base for researching new treatment methods and nerve repair after optic nerve and central nerve injuries. Further studies are planned to evaluate the clinical significance of CNS regeneration using this model system.

## **Material and Methods**

### Measurement of the Diameters of Rat Optic Nerves and Manufacture of the Semi-transected Optic Nerve Quantitative Amputator

Wistar rats used in the experiments (n = 20, 8–10 week-old, 200–250 g) were housed at 21–23°C on a 12-h light/dark cycle (lights on at 8:00 am). Food and water were freely available. Rats were anesthetized with 10% (100 mL/L) chloral hydrate (i.p., 3.5 mL/kg). The animals were perfused through the ascending aorta with (40 g/L) 4% paraformaldehyde (PFA) in 0.1 M phosphate-buffered saline (PBS, pH 7.4). Whole eyeballs and optic nerves were removed on both sides, with optic nerve sheaths uncovered, and snap-frozen in liquid nitrogen. Thirty minutes later, the samples were removed from liquid nitrogen and the diameters of optic nerves (n = 40), 2.0 mm behind both eyeballs were measured with a spiral micrometer (Links, China). The mean diameter of the transverse sections was 0.69±0.06 mm at 2.0 mm behind both eyeballs.

The semi-transected optic nerve quantitative amputator is made of stainless steel. The main parts of the amputator are as follows: frontal harness hole, cutting groove, connecting rod and handle. The size of the frontal harness hole of the amputator is dependent on the mean value of the optic nerve diameter measured and the depth of the cutting groove is half the mean value of the optic nerve diameter (Figure 1A). All procedures involving the use of animals in this study complied with the regulations and protocols of the Ethic Committees of Harbin Medical University and the Guide for the Care and Use of Laboratory Animals of the US National Institutes of Health (NIH Publication No.85–23, revised 1996). Animal care and experimental procedures were specifically approved by the Animal Experimental Committee of Harbin Medical University.

### Surgical Procedures

Anesthetized rats were de-furred and their skin was prepared and sterilized. The right eye was prepared for surgery as the experimental eye and the left eye was the non-operative control. Under binocular operation microscope (Leica M520 F40, Germany), an incision of 5.0 mm was made backward and horizontally along the skin of the outer canthus; the palpebra superior was suspended fixedly outward and upward; the conjunctiva in fornix was cut open, followed by blunt dissection from the outer-upper quadrant between the superior rectus and the lateral rectus to behind the eyeball. The optic nerve meninges were slivered with a 2.0 mm opening and the optic nerve was exposed. The harness hole of the amputator was fixed to the optic nerve 2.0 mm behind the eyeball, within the optic nerve meninges. The upper half of the optic nerve was removed along the cutting groove of the amputator perpendicular to the horizontal axis. Finally, the semi-transection was completed (Figure 1B).

After surgery, the conjunctiva and skin were sutured, the wound was sterilized, Chloromycetin eye drops were added into the experimental eye and erythromycin ophthalmic ointment was applied to the wound and eyeball. No treatment was conducted to the left eye, which served as the control. After surgery, the rats were housed in different cages and their experimental eyes were treated with Chloromycetin eye drops and erythromycin ophthalmic ointment for the first three successive days (Figure 1C–E).

### Retrograde Labeling of RGCs and Axons

**Figure 11:**
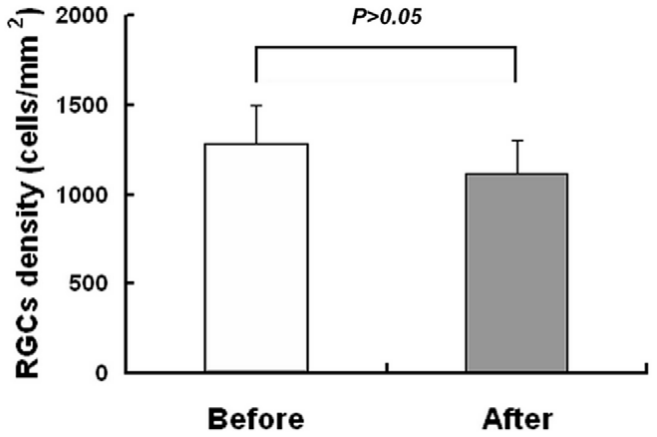
Comparison of the RGCs density resulted from the two FG-labeling methods. “Before” is the group that FG was applied 7 days before semi-transection (n = 6). “After” is the group that FG was applied right after the semi-transection (n = 6). In both groups, rats were sacrificed 7 days after the injury. Error bars indicate SD.

In the preliminary experiment, FG was applied to retro-label the RGCs between two groups individually at different time - in one group FG was applied 7 days before the semi-transection; in the other group FG was applied immediately after the semi-transection and then the animals were sacrificed 7 days after applying FG. Both groups were allowed to survive 7 days after the injury. The result showed that there was no significant difference in RGC density between the two groups (Figure 11). Wang et al. and Levkovitch-Verbin et al. reported that if FG is injected for several weeks, it will be taken up by macrophages and/or microglias that may lead to the number of counted fluoresce points are more than original survived RGCs population [11], [21]. To avoid over-counting the RGCs, FG was applied 1 week before the rats were euthanized[42]–[44]. Briefly, Wistar rats (n = 30, 8–10 week-old, 200–250 g) were divided into 5 groups randomly (6 rats of each group) according to the survival time after surgery (1 week (F1W), 2 weeks (F2W), 4 weeks (F4W), 8 weeks (F8W), 12 weeks (F12W)). Rat optic nerve from F1W rats were retrograde labeled immediately after semis-transection. The remaining groups were done 7 days before killing. The anesthetized rats were fixed in the stereotactic apparatus (STOELTING, USA), de-furred and their skin was sterilized. The skin was cut open along the middle line of the calvarium; the sagittal suture and lambdoid suture were disclosed to locate the Bregma point. Four small holes were drilled at the parietal bone according to the Paxinos stereotaxic atlas [45]. Rats were then implanted with a microinjection probe at the position of the bilateral superior colliculi (AP −6.8 mm; LR ±1.6 mm; DV −4.0 mm, from Bregma) and lateral geniculate body (AP −4.6 mm; LR ±4.0 mm; DV −5.6 mm, from Bregma). Four percent Fluorogold (Biotium, USA) 2 µl, dissolved in 0.1 M PBS (pH 7.4) with 10% dimethyl sulfoxide and 1% Triton X-100 was microinjected into the bilateral superior colliculi and lateral geniculate body. The needle was removed after 10 minutes. After hemostasis, the exocranium and skin were sutured and antibiotic ointment was applied to the wounds. The rats were housed in different cages after surgery.

### RGC Counting

After being labeled with FG for 7 days, the rats were anesthetized with 10% (100 mL/L) chloral hydrate (i.p., 3.5 mL/kg) at the end of weeks 1, 2, 4, 8 and 12 after optic nerve semi-transection. The rats were perfused through the ascending aorta with the mixture of 4% PFA and 0.1 M PBS (pH 7.4). The eyes were labeled with one stitch at the superior rectus, followed by the removal of both eyeballs and the affiliated optic nerves, the optic nerves were severed. The eyeball was post-fixed for 1 hour in 4% PFA and 0.1 M PBS (pH 7.4) mixture in darkness. Subsequently, the cornea and the corporis vitre were removed, the retinas were carefully isolated from the sclera under microscopy, and the retina was positioned according to the labeled point of the suture line. The retina was divided into four parts (four quadrants), taking the optic disc as the center, dorsal-ventral and rostral-caudal direction as coordinate axis. The retina was dissected along dorsal-ventral and rostral-caudal direction and flat-mounted on a newly gelatinized glass slide with the retinal nerve fiber layer facing up. Finally, the retina was covered with 75% glycerine.

The retina was observed under confocal microscopy (Nikon TE-2000E; Nikon Corp., Japan) within 2 hours after sampling. Sampled RGCs are used by most researchers to estimate total RGCs [16], [46]. Three observation areas were selected at a distance of 1/6, 1/2, and 5/6 of the retina radius from the optic nerve disc at each radius quadrant. Twelve fluorescent photos were taken under 200× magnification and the visual field size for each observation area was 0.3183 mm×0.3183 mm. The FG-labeled RGCs in every visual field was counted directly and the mean values of the labeled RGC numbers in 12 visual fields were calculated. Cell counts were performed according to a double-blind method by three different investigators. The density of RGCs was equal to cell count/area in every visual field; RGC survival rate that was the percentage of the surviving RGC densities after injury versus the control RGC densities at the same time point was calculated.

RGC apoptosis rate was calculated at each testing time-point (weeks 1, 2, 4, 8 and 12) after semi-transection by the following formula: the mean of RGC apoptosis rate per week = [(RGC density of the experimental eyes at previous testing time-point – RGC density of the experimental eyes at subsequent testing time-point)/the mean RGC density of the control eyes] ÷ time duration (number of weeks).

### Optic Nerve Sectioning

The dissected optic nerves were post-fixed for 6–8 h in 4% PFA in 0.1 M PBS (pH 7.4). After being washed with 0.1 M PBS, the optic nerves were dehydrated in 30% sucrose (4°C) overnight. The tissues were removed and the optic nerves at 1.0 mm behind the eyeball were enveloped by OCT gelatin, followed by serial sectioning at the transection of the optic nerve (the thickness of each section was 10 µm). The sections of nerves were flattened onto the newly-gelatinized slides and the fluorescence and distribution of the labeled nerve fibers were observed under confocal microscopy (100×) (Nikon TE-2000E; Nikon Corp., Japan).

### Examination of Visual Evoked Potential (VEP)

Wistar rats (n = 30, 8–10 week-old, 200–250 g) were randomly divided into 5 groups (6 rats for each group) depending on the testing time-point after semi-transection of the optic nerve (3 days (V3D), 1 week (V1W), 2 weeks (V2W), 4 weeks (V4W), 8 weeks (V8W)). The pupils were dilated with Mydrin (Santen, Japan), after being anesthetized with 10% chloral hydrate (i.p. 3.5 mL/kg). Pattern visual-evoked potential (P-VEP) was recorded with Electromyograph and Evoked Potential Equipment (Keypoint 4, Denmark) in a dark-adapted condition at different testing time-points. The degree of anesthesia was the same when the visual electrophysiological tests were performed in rats. The recording electrodes (stainless needle) were inserted into the subperiosteum of the external occipital protuberance at the middle of the line between the two ears. The reference electrodes were inserted under the subperiosteum of the nose root and the ground electrodes were placed in the tails of the rats (impedance<1.6 kΩ). The visual stimulus of checker turnover (black and white) in full visual fields was used, with a size of 12×16, brightness of 60 cd/m^2^, contrast of 80%, Sweep 30ms/D, Sens 5uV/D, stimulation frequency of 3 Hz, low frequency of band pass of 1 Hz, high frequency of 1 kHz; superposition was conducted for 200 times. Stable waveforms were recorded 3 different times in each eye and the contralateral eyes were shaded with an eyeshade when the detected eyes were tested. The waveform recorded by P-VEP was stable N_70_-P_100_-N_145_ wave, which consisted of three major components, the first small positive peak is N_70_, the second large negative peak is P_100_, and the third small positive peak is N_145_. The parameters observed were latency of P_100_ (P_100_ wave response time from signal stimuli, ms) and amplitudes of N_70_, P_100_, N_145_ (µV). All the parameters values were measured automatically by computer output and average of the three measurements were calculated. The amplitudes of N_70_-P_100_ (from N_70_ wave peak to P_100_ wave trough, µV) and P_100_-N_145_ (from P_100_ wave trough to N_145_ wave peak, µV) were calculated as well.

The increasing ratio of P_100_ latency for each animal was calculated as follows: the increasing ratio of P_100_ latency = (P_100_ latency of the experimental eye – P_100_ latency of the control eye)/P_100_ latency of the control eye × 100%. The decreasing ratio of N_70_-P_100_ amplitude [decreasing ratio of N_70_-P_100_ amplitude = (N_70_-P_100_ amplitude of the control eye – N_70_-P_100_ amplitude of the experimental eye)/N_70_-P_100_ amplitude of the control eye × 100%] and P_100_-N_145_ amplitude [decreasing ratio of P_100_-N_145_ amplitude = (P_100_-N_145_ amplitude of the control eye – P_100_-N_145_ amplitude of the experimental eye)/P_100_-N_145_ amplitude of the control eye × 100%] were also calculated.

### Pupillary Reflex Observation before and after Semi-transection

Before anesthetization and semi-transection, both eyes (n = 24) of rats (n = 12) in the V8W and F12W groups were observed and the pupillary diameters were recorded. After semi-transection, the papillary diameters of both eyes were examined and recorded immediately, for three successive days and then at least once a week until week 8.

### Pupillary Reflex Observation before and after FG Retrograde Labeling

The modification of pupillary diameter of rats in F1W group were observed and recorded before and after FG was injected into the bilateral superior colliculi and lateral geniculate body.

### Statistics

The data were processed with SPSS 11.0 software and the means and standard deviations were calculated. The analysis of variance was used to compare the difference between the control and experimental eyes at each time point and difference among the control eyes at different time points. Data are expressed as means ± SD, and values of *P*<0.05 were considered statistically significant.
